# Thymic Stromal Lymphopoietin Is Critical for Regulation of Proinflammatory Cytokine Response and Resistance to Experimental *Trypanosoma congolense* Infection

**DOI:** 10.3389/fimmu.2017.00803

**Published:** 2017-07-14

**Authors:** Chukwunonso Onyilagha, Rani Singh, Abdelilah Soussi Gounni, Jude Ezeh Uzonna

**Affiliations:** ^1^Faculty of Health Sciences, Department of Immunology, College of Medicine, University of Manitoba, Winnipeg, MB, Canada; ^2^Faculty of Health Sciences, Department of Medical Microbiology, College of Medicine, University of Manitoba, Winnipeg, MB, Canada

**Keywords:** *Trypanosoma congolense*, infection, thymic stromal lymphopoietin, cytokines, immunosuppression

## Abstract

African trypanosomiasis (sleeping sickness) poses serious threat to human and animal health in sub-Saharan Africa. Because there is currently no vaccine for preventing this disease and available drugs are not safe, understanding the mechanisms that regulate resistance and/or susceptibility to the disease could reveal novel targets for effective disease therapy and prevention. Thymic stromal lymphopoietin (TSLP) plays a critical role in driving Th2 immune response. Although susceptibility to experimental *Trypanosoma congolense* infection in mice is associated with excessive proinflammatory responses due in part to impaired Th2 response, the role of TSLP in resistance to African trypanosomiasis has not been well studied. Here, we investigated whether TSLP is critical for maintaining Th2 environment necessary for survival of *T. congolense*-infected mice. We observed an increased TSLP level in mice after infection with *T. congolense*, suggesting a role for this cytokine in resistance to the infection. Indeed, TSLPR^−/−^ mice were more susceptible to *T. congolense* infection and died significantly earlier than their wild-type (WT) controls. Interestingly, serum levels of IFN-γ and TNF-α and the frequency of IFN-γ- and TNF-α-producing CD4^+^ T cells in the spleens and liver were significantly higher in infected TSLPR^−/−^ mice than in the WT control mice. Susceptibility was also associated with excessive M1 macrophage activation. Treatment of TSLPR^−/−^ mice with anti-IFN-γ mAb during infection abolished their enhanced susceptibility to *T. congolense* infection. Collectively, our study shows that TSLP plays a critical role in resistance to *T. congolense* infection by dampening the production of proinflammatory cytokines and its associated M1 macrophage activation.

## Introduction

African trypanosomiasis, also called sleeping sickness in human, is caused by blood parasites belonging to the genus *Trypanosoma*. The disease continues to pose great danger to the health of human and livestock in the affected regions and threatens over 60 million people in 36 different countries in sub-Saharan Africa (1). The animal form of the disease is associated with massive agricultural and economic problems, and it has been suggested that elimination of the disease in livestock would save Africa an estimated $4.5 billion per year (2). Among the several species of trypanosomes that cause disease in animals in Africa, *Trypanosoma congolense* is considered the most important pathogen especially for cattle (3). There is currently no vaccine available to prevent the disease because of inadequate information regarding the factors that regulate resistance and susceptibility to the infection, and the parasite’s ability to evade the host immune response through antigenic variation (4).

Antigen-specific B cell (antibody) responses are critical for control of *T. congolense* infection (5–7), as these parasites are completely extracellular in nature. *T. congolense*-specific antibodies are able to opsonize the parasites leading to phagocytosis and clearance by macrophages (mostly by kupffer cells) (8). Previous report associated the production of IgG2a and IgG3 (but not IgM) antibodies with more effective parasite clearance and resistance to *T. congolense* infection in mice (6). Similarly, increased IgG1 antibody level has also been associated with resistance in *T. congolense* infected cattle (9). In contrast, the expansion of CD4^+^CD25^+^Foxp3^+^ regulatory T cells (Tregs) (10–12) and excessive release of proinflammatory cytokines during infection enhance host susceptibility to infection (13, 14).

During infection with African trypanosomes, classically activated macrophages (M1) contribute to parasite clearance through phagocytosis (8), release of proinflammatory cytokines and nitric oxide (13, 15, 16). Activated macrophages also present trypanosomal antigens to CD4^+^ T cells (17) leading to more activation and production of cytokines by CD4^+^ T cells (17). Because macrophages expand and continue to carry out their functions in the spleen and liver after infection, they often get over-activated, releasing excessive amounts of proinflammatory molecules that eventually contributes to disease severity and death of infected mice (13, 14). In contrast, because of their anti-inflammatory properties (18), alternatively activated macrophages (M2) play a crucial role in dampening inflammatory responses during the advanced stage of infection with African trypanosomes (19, 20). Although overproduction of IFN-γ and other proinflammatory cytokines has been linked to the death of infected susceptible mice (13, 14), IFN-γ plays protective roles early in infection by helping in the production of nitric oxide and parasite-specific immunoglobulins (7, 21, 22), which are required for optimal immunity during infection.

Thymic stromal lymphopoietin (TSLP) is a cytokine that plays critical role in driving Th2 differentiation (23–25) and promoting B cell development (26, 27). Its receptor (TSLPR) is expressed on various important cell types including T, B, dendritic and epithelial cells (28, 29). Although the role of TSLP in immunity has been well studied in allergic diseases (30–33), its role in resistance to parasitic diseases including African trypanosomiasis is still poorly defined. Although an early Th1 immune response is necessary to clear trypanosome parasites during the early stage of infection (6), the inability of infected C57BL/6 mice to switch from Th1 to Th2 immune response as well as from classically activated macrophages (M1) to alternatively activated macrophages (M2) has been linked to death of infected mice (34, 35). Furthermore, reduction in nitric oxide production and elevated IL-10 and IL-4 mRNA levels were associated with resistance in *T. congolense* infected cattle (9, 36). These observations suggest the need to further investigate the role of Th2 immune response in the pathogenesis of *T. congolense* infection. It is conceivable that a Th2 response during the late stage of infection would help to curb the excessive inflammatory responses observed during *T. congolense* infection and prolong the life of infected animals (37). Because TSLP-TSLPR engagement and signaling have been shown to promote polarization of macrophages into M2 phenotype (38) and M2 macrophages regulate the activation of inflammatory M1 macrophages in trypanosome-infected animals (19), we hypothesized that TSLP would be critical for survival during the late stage of infection by limiting excessive inflammatory responses in infected animals (37).

Here, we show that TSLP levels are increased in the serum after infection of mice with *T. congolense* and deficiency of TSLP signaling as seen in TSLPR^−/−^ mice results in susceptibility during the chronic phase of *T. congolense* infection in mice. This susceptibility was associated with excessive production of proinflammatory cytokines including IFN-γ and TNF-α and enhanced M1 macrophage activation. Treatment of infected TSLPR^−/−^ mice with anti-IFN-γ abolished their enhanced susceptibility, reduced the levels of proinflammatory cytokines in the serum, and rescued these mice from early death.

## Materials and Methods

### Ethics Statement

The experiments described here were approved by the University of Manitoba Animal Care Committee and carried out in accordance with the regulation of the Canadian Council on Animal Care (Protocol Number 14-014).

### Mice

Six- to eight-week-old female C57BL/6 mice and CD1 (outbreed Swiss white mouse) used in all the experiments described here were purchased from the Central Animal Care Services (CACS), University of Manitoba, Winnipeg, MB, Canada. The origin and phenotype of TSLPR^−/−^ mice have been previously described (39). TSLPR^−/−^ mice on C57BL/6 background were bred by the CACS and supplied when required. The housing and maintenance of all experimental animals were carried out according to the recommendations of the Canadian Council of Animal Care.

### Parasite and Infection of Mice

In all the experiments described here, *T. congolense* (Trans Mara strain), variant antigenic type TC13 was used. The origin of this parasite strain has been previously described (40). To prepare parasites for infection, CD1 mice were immunosuppressed intraperitoneally by injection of cyclophosphamide (Cytoxan; 200 mg/kg). After 48 h, TC13 stabilates (40) were intraperitoneally injected into these mice. Three days after infection, the mice were anesthetized using isoflurane and blood was collected by cardiac puncture. Parasites were purified from the blood using DEAE-cellulose anion-exchange chromatography (41). Eluted parasites were washed in Tris-saline glucose (TSG), counted, resuspended in TSG containing 10% heat-inactivated FBS, and diluted to the desired concentration of 10^4^/mL before infection. Infection of mice was done intraperitoneally with 100 µL of this dilution (containing 10^3^ parasites).

### Estimation of Parasitemia and Anti-IFN-γ Treatment

To estimate parasitemia, a drop of blood taken from the tail vein of each *T. congolense*-infected mouse on a microscopic slide was covered with cover slip, and parasitemia was determined by counting the number of parasites presents in at least 10 fields at 400× magnification of light microscope. During periods of heavy parasite load, estimation was done as described previously (42). For experiments requiring anti-IFN-γ treatment, mice were injected intraperitoneally with anti-mouse IFN-γ, clone XMG1.2, or control Ig (BioXcell) (1 mg/mouse).

### Direct *Ex Vivo* Staining and Flow Cytometry

Mice were sacrificed at indicated days after infection and the spleens and liver were harvested and processed into single-cell suspensions. Liver cells were resuspended in 40% percoll, layered above 70% percoll, and centrifuged at 750 *g* for 20 min at 22°C. The interface containing the lymphocytes was carefully collected. All cells were washed two times with PBS, resuspended at final concentration of 4 × 10^6^/mL in complete tissue culture medium (DMEM supplemented with 10% heat-inactivated fetal bovine serum, 2 mmol l-glutamine, 100 U/mL Penicillin and 100 µg/mL streptomycin). Cells were directly stained *ex vivo* for CD4 and CD25 expression and intracellularly for Foxp3 using the Tregs staining kit (eBioscience, San Diego, CA, USA) in accordance with the manufacturer’s recommendations. In some experiments, cells were stimulated with phorbol myristic acetate (50 ng/mL), ionomycin (500 ng/mL), and brefeldin A (10 µg/mL) for 4 h and stained for CD4 surface expression and for intracellular cytokine (TNF-α, IFN-γ, and IL-10) expression. The germinal center B cell response was assessed by staining splenic cells with fluorochrome-conjugated antibodies against B220, GL7, and Fas (eBiosciensce). To assess M1 and M2 activation during infection, antibodies against surface CD11b and F4/80 (eBioscience) were used; cells were further fixed, permeabilized, and stained with antibodies against inducible nitric oxide synthase (iNOS), MMR (eBioscience), and Arginase 1 (Arg1, R&D Systems). After staining, all samples were washed routinely in FACS buffer, acquired using BD FACS Canto II cytometer (BD Bioscience, San Diego, CA, USA), and analyzed using FlowJo software (BD Bioscence).

### Immunofluorescence Microscopy

The spleens from infected mice were collected on indicated days and embedded in OCT (Tissue-Tek, Torrance, CA, USA) before being snap frozen in liquid nitrogen. The frozen sections were cut to 8–10 µm size and fixed with paraformaldehyde for 15 min, washed with PBS, and air-dried. Sections were blocked for 30 min with mouse Fc Block, washed with PBS containing Tween 20 (0.05%) and stained for 1 h at room temperature with antibody cocktail-containing FITC-labeled PNA (Vector laboratories, Burlington, ON, Canada), PE-labeled anti-CD4, and APC-labeled anti-IgD (BD Biosciences). The slides were mounted in Prolong Gold anti-fade reagent (Molecular Probes) after washing with PBS and viewed with Zeiss AxioObserver Spinning Disk Confocal Microscope.

### Serum Collection, ELISA, and Measurement of Trypanosome-Specific Antibodies

Mice were anesthetized using isoflurane and blood was collected by cardiac puncture using a 1-mL syringe and 25-G needle. Blood samples were kept at 4°C for 4 h and spun at 2,400 rpm for 10 min, and serum was collected and stored at −20°C until used for antibody determination. Serum levels of *Trypanosome*-specific IgM and IgG antibodies in infected mice were determined by ELISA as previously described (6, 43). The levels of IFN-γ, TNF-α, and IL-10 in the serum were determined by sandwich ELISA using antibody pairs purchased from BD Biosciences according to the manufacturer’s suggested protocols. The sensitivities of the cytokine ELISAs range from 7.5 to 31 pg/mL. Mouse TSLP ELISA Kit was purchased from SIGMA-ALDRICH, and the TSLP levels were determined by ELISA according to the manufacturer’s suggested protocol.

### Cell Isolation and Real-time Polymerase Chain Reaction

At indicated days after infection, mice were sacrificed and the spleens and livers were harvested and processed into single-cell suspensions in complete tissue culture medium. Spleen cells were washed, lysed with RBC lysis buffer, and resuspended in complete tissue culture medium before macrophage purification. The livers were perfused (right ventricle) with 10 mL of ice-cold-PBS, digested with collagenase-D (125 µg/mL) for 30 min at 37°C, and homogenized in complete tissue culture medium. The cells were then passed through a 70-µm cell strainer (VWR, Mississauga, ON, Canada) and washed with 30 mL Hanks balanced salt solution (HBSS) (Invitrogen, ON, Canada) at 1,200 rpm for 5 min. Red blood cells were lysed with RBC lysis buffer and washed with HBSS before re-suspending in 4 mL of 40% percoll (Sigma). Lymphocytes were separated by layering the cells on top of 70% percoll and spinning (without brakes) at 750 *g* at 22°C for 20 min. The interface that contains the mononuclear cells was gently collected, washed and re-suspended in complete tissue culture medium. Enrichment of CD11b^+^ cells was carried out using AutoMACS positive selection kit (Miltenyi Biotec), with over 95% of the enriched cells being F4/80^+^ when assessed by flow cytometry. Total RNA extraction from enriched spleen and liver macrophages was carried out using TRIzol (Invitrogen, USA), and quantified using Nano-Drop spectrophotometer (Thermo Scientific). cDNA was synthesized using high capacity cDNA Reverse Transcription Kit (Applied Biosystems). Real-time PCR was performed using SYBR Green master mix (Applied Biosystems) and the expressions of Arg1 and YM1 were analyzed. Changes in relative gene expression were normalized to Eukaryotic Translation Elongation Factor 2 (Eef2). The specific primer sequences used are: YM1: CAGGTCTGGCAATTCTTCTGAA (forward) and GTCTTGCTCATGTGTGTAAGTGA (reverse). Arginase 1: TTGGGTGGATGCTCACACTG (forward) and GTACACGATGTCTTTGGCAGA (reverse). Eef2: TGTCAGTCATCGCCCATGTG (forward) and CATCCTTGCGAGTGTCAGTGA (reverse).

### Statistics

All data presented here are represented as mean and SEM. Two-tailed Student’s *t*-test or ANOVA were used to compare means and SEM between two groups using GraphPad Prism software. Differences were considered significant at *p* < 0.05.

## Results

### TSLPR^−/−^ Mice Succumb to Chronic *T. congolense* Infection

**Figure 1:**
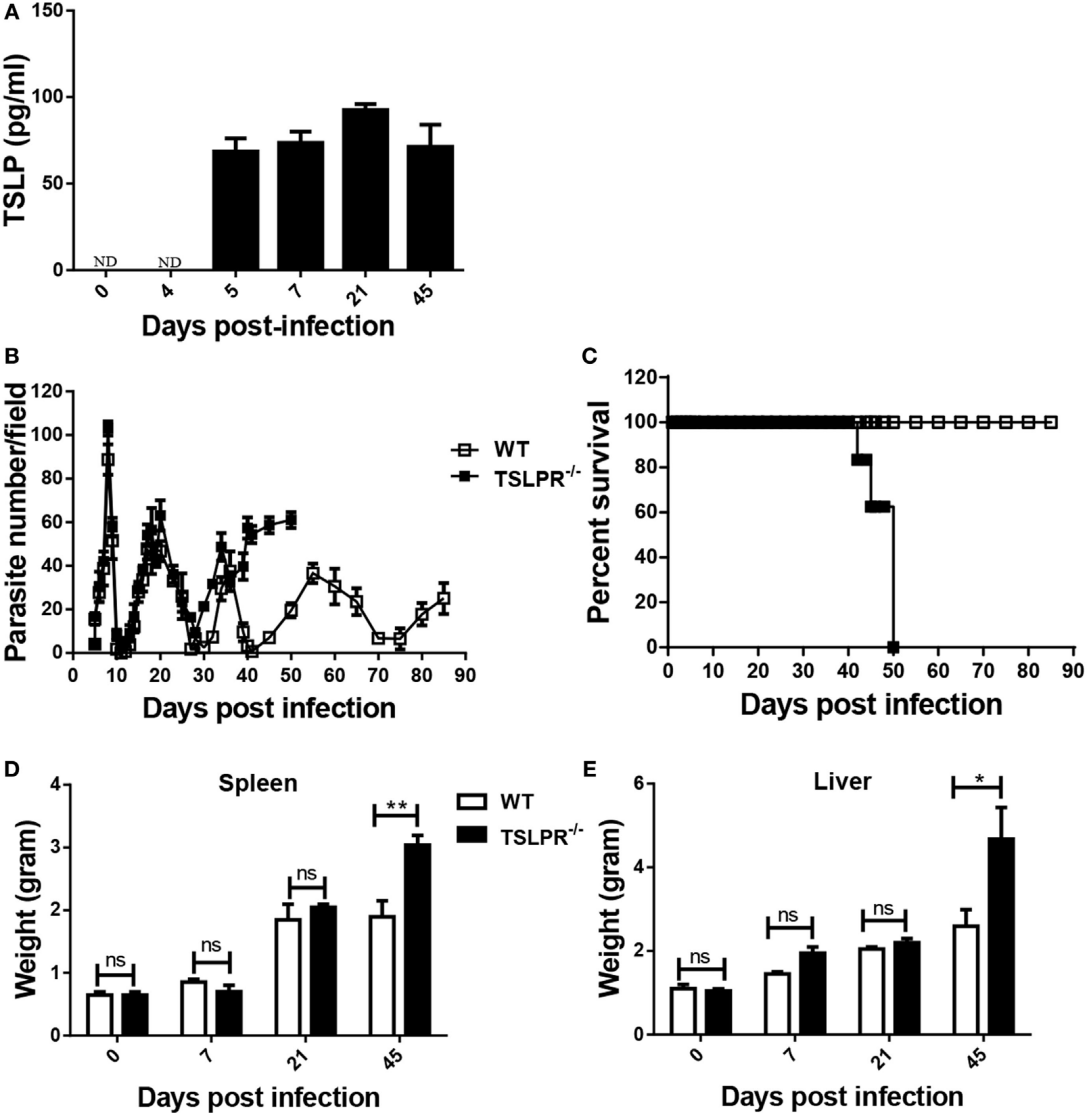
Thymic stromal lymphopoietin (TSLP) signaling is critical for optimal resistance to *Trypanosoma congolense* infection in mice. Groups of wild-type (WT) and TSLP receptor deficient (TSLPR^−/−^) C57BL/6 mice were infected intraperitoneally with 10^3^ *T. congolense*, clone TC13 and serum levels of TSLP were determined by ELISA (in WT only) at the indicated times **(A)**. In addition, parasitemia **(B)** was also estimated daily by direct counting of parasites in tail blood smears at 400× magnification and mice were monitored over time to determine the percentage survival **(C)**. At indicated times, some infected mice were sacrificed and spleen **(D)** and liver **(E)** weights determined. The results presented are representative of three different experiments (*n* = 4–5 mice per group) with similar outcomes. ns, not significant; *p < 0.05; **p < 0.01.

A dominant Th2 response has been associated with resistance to experimental African trypanosomiasis in mice (9, 36) especially during the late stage of infection. Given that TSLP drives Th2 response (23–25), we hypothesized it would play a critical role in resistance to *T. congolense* infection in mice. First, we assessed the level of TSLP in serum of mice after infection with *T. congolense*. We found increased level of TSLP in the serum of mice infected with *T. congolense* (Figure 1A) suggesting a possible role of this cytokine during infection.

To directly assess the role of TSLP in resistance to this parasite, we infected WT and TSLPR^−/−^ mice with 10^3^ *T. congolense* and monitored parasitemia and survival. There was no significant difference in the prepatent period and level of parasitemia during the early phase of the infection between WT and TSLPR^−/−^ mice (Figure 1B). However, TSLPR^−/−^ mice developed fulminating parasitemia during the chronic phase and succumbed to the infection (mean survival time 48 ± 8 days, Figure 1C) unlike their WT control mice that survived with lower parasitemia until day 85 when the experiment was terminated. The enhanced susceptibility of TSLPR^−/−^ mice was associated with increased hepatosplenomegaly (Figures 1D,E), a pathological feature commonly associated with increased organ damage, cellular activation, and expansion of the reticuloendothelial system (44, 45). Of note is the fact that the hepatosplenomegaly coincided with the onset of uncontrolled parasitemia in the late phase of the infection in TSLPR^−/−^ mice (see Figure 1B). Interestingly, there was no difference in serum levels of ALT and AST (not shown) between infected WT and TSLPR^−/−^ mice, suggesting that increased organ pathology resulting from hepatosplenomegaly may not be directly responsible for the enhanced susceptibility of infected TSLPR^−/−^ mice.

### Serum Trypanosome-Specific Antibody Levels and Germinal Center Reaction in Infected TSLPR^−/−^ Mice

**Figure 2:**
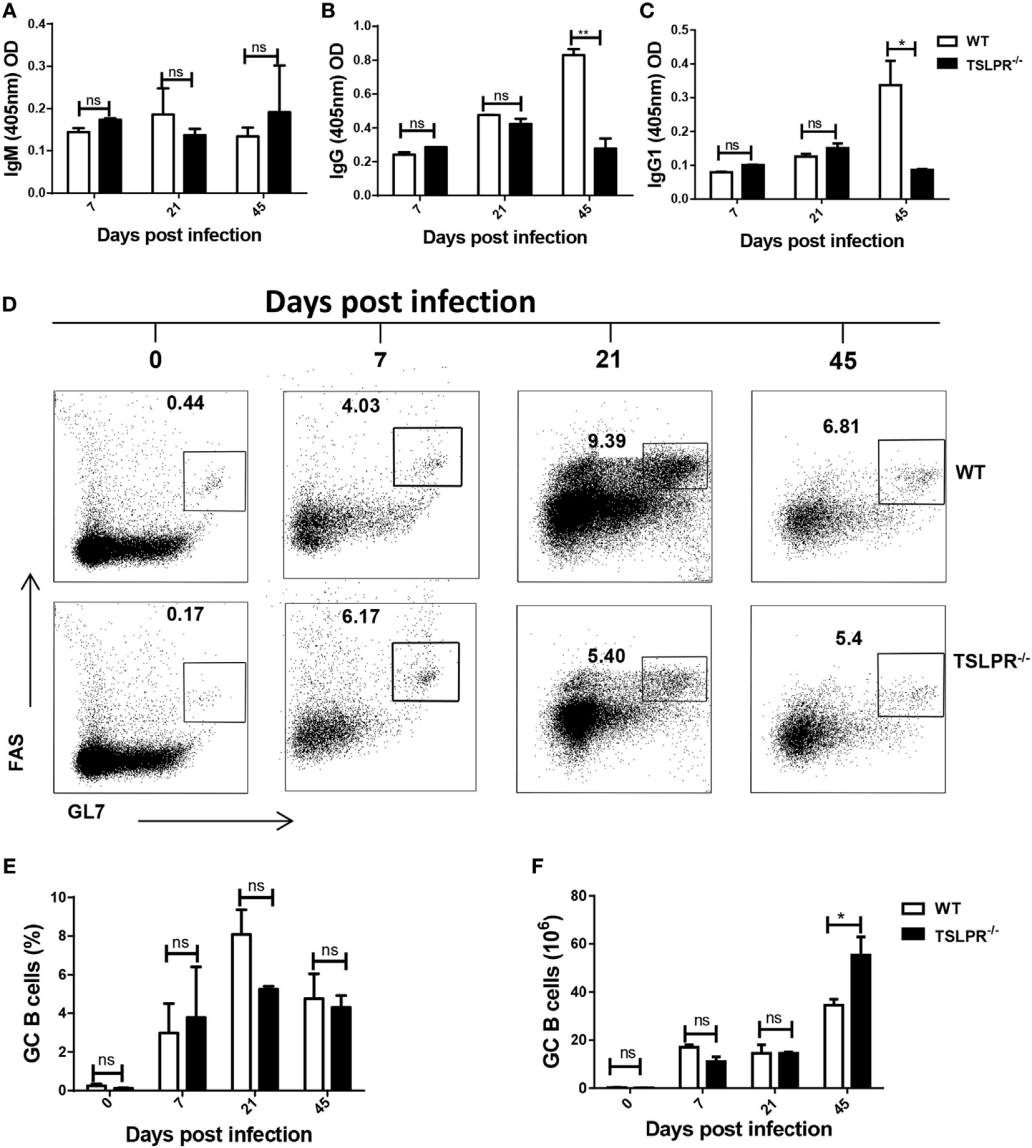
Serum trypanosome-specific antibody levels and germinal center B cell response in infected TSLPR^−/−^ mice. Groups of wild-type and TSLPR^−/−^ mice infected with 10^3^ *Trypanosoma congolense* were sacrificed at indicated times and serum levels of trypanosome-specific IgM **(A)**, IgG **(B)**, and IgG1 **(C)** antibodies were determined by ELISA. In addition, the percentages **(D,E)** and absolute numbers **(F)** of germinal center B cells (B220^+^GL7^+^FAS^+^) in the spleens of infected mice were assessed directly *ex vivo* by flow cytometry. Cells were first gated for B220 expression and then assessed for GL7 and FAS expression. The results presented are representative of two different experiments (*n* = 4–5 mice per group) with similar outcomes. ns, not significant; *p < 0.05; **p < 0.01.

Because antibodies (especially IgG and its subclasses) have been shown to be important in *T. congolense* control (5–7), and because TSLP has been reported to influence IgG1 production (46), we investigated whether the enhanced susceptibility of TSLPR^−/−^ mice was related to impaired antibody response to the parasites. We infected WT and TSLPR^−/−^ mice with *T. congolense* and assessed serum levels of parasite-specific IgM and IgG antibodies at different time points. Results show that there was no difference in the kinetics and magnitude of IgM antibody responses in infected WT and TSLPR^−/−^ mice (Figure 2A). However, there was significantly reduced serum levels of trypanosome-specific IgG (Figure 2B) and IgG1 (Figure 2C) but not IgG2a and IgG3 (not shown) in the TSLPR^−/−^ mice later in the infection. This defective IgG antibody response corresponds to the onset of uncontrolled parasitemia in these mice and suggests that the ability to mount effective germinal center response in these mice may be impaired. However, flow cytometric analysis shows no significant difference in either the percentages (Figures 2D,E) or absolute numbers (Figure 2F) of germinal center B cells in the spleens of infected WT and TSLPR^−/−^ mice at different time points after infection. However, by day 45 postinfection, there was a significant increase in the absolute numbers of germinal center B cells in infected TSLPR^−/−^ mice, which could be related to the higher splenomegaly observed during the late stage of infection in TSLPR^−/−^ mice (see Figure 1D). Immunofluorescence staining of the germinal center structure in the spleens of infected mice further confirmed the absence of significant difference in germinal center response between TSLPR^−/−^ and WT mice (Figure S1 in Supplementary Material).

### Level of Tregs in Infected TSLPR^−/−^ Mice

**Figure 3:**
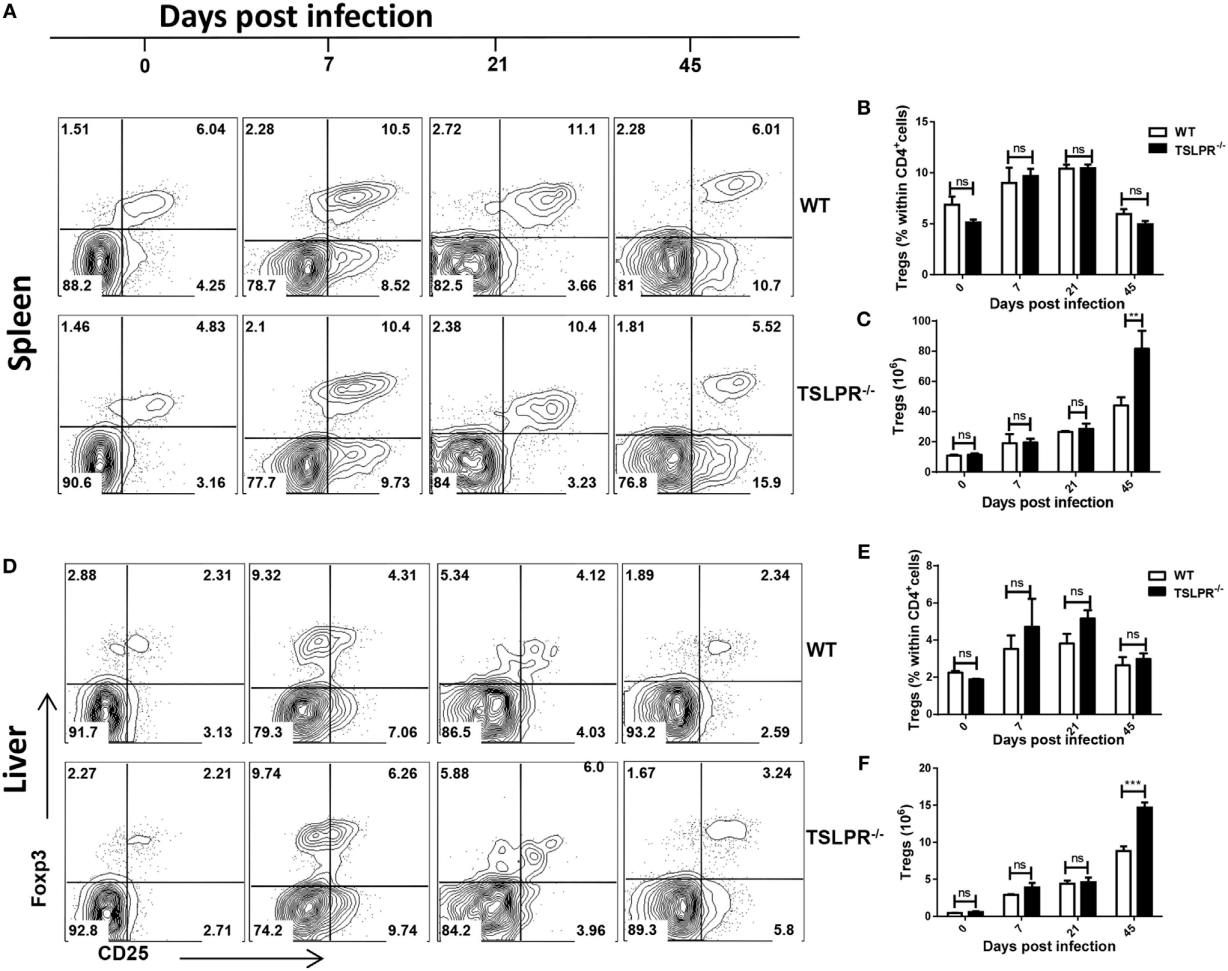
Comparable numbers of regulatory T cells (Tregs) in infected wild-type (WT) and TSLPR^−/−^ mice. Infected WT and TSLPR^−/−^ mice were sacrificed at indicated times and their spleen and liver cells were directly stained *ex vivo* for surface expression of CD4 and CD25 and intracellularly for Foxp3 expression. Shown are contour plots **(A,D)** and bar graphs **(B,C,E,F)** of percentages **(A,B,D,E)** and absolute numbers **(C,F)** of CD4^+^CD25^+^Foxp3^+^ cells in the spleens **(A–C)** and liver **(D–F)** at different times after infection. Cells were first gated for CD4 expression and then assessed for CD25 and Foxp3 expression. The results presented are representative of two different experiments (*n* = 4–5) with similar outcomes. ns, not significant; **p < 0.01; ***p < 0.001.

Several reports have shown that CD4^+^CD25^+^Foxp3^+^ Tregs play disease-promoting role in experimental African trypanosomiasis (10–12). Since TSLPR^−/−^ mice were more susceptible to *T. congolense* infection than their WT counterpart mice, we assessed the numbers of Treg cells in the spleens and liver at different times after infection. As shown in Figure 3, there was no difference in the percentages (Figures 3A,B,D,E) and absolute numbers (Figures 3C,F) of Tregs in the spleens (Figures 3A–C) and Liver (Figures 3D–F) of infected WT and TSLPR^−/−^ mice at different times after infection. The increase in the absolute numbers of Tregs in infected TSLPR^−/−^ mice on day 45 after infection (Figures 3C,F) could be explained by the higher hepatosplenomegaly observed during the late stage of infection (see Figures 1D,E) in TSLPR^−/−^ mice. These results show that the enhanced susceptibility of TSLPR^−/−^ mice to *T. congolense* infection is not related to differences in Treg numbers.

### Enhanced Levels of IFN-γ and TNF-α Levels in Infected TSLPR^−/−^ Mice

**Figure 4:**
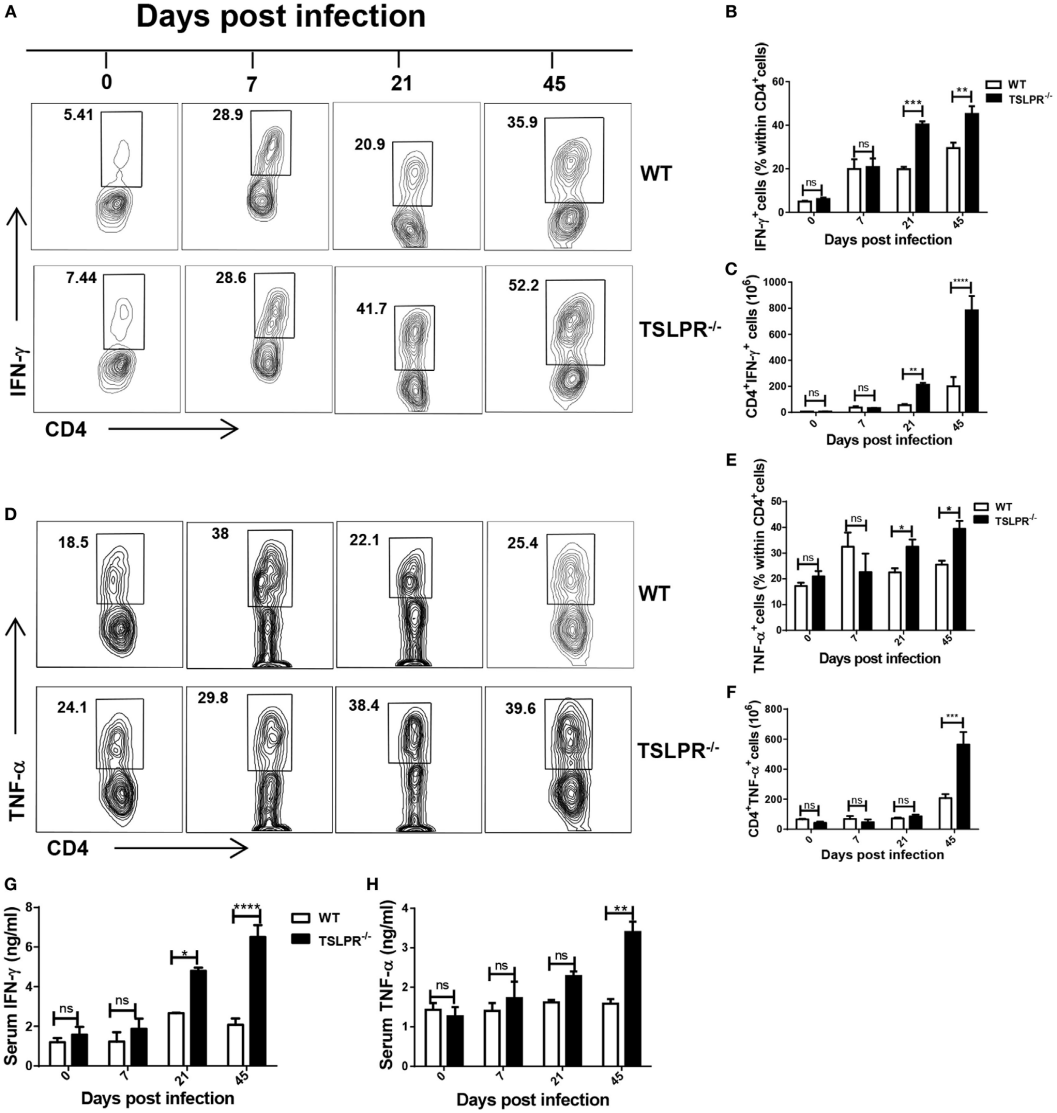
Elevated serum levels and numbers of IFN-γ- and TNF-α-producing CD4^+^ T cells in infected TSLPR^−/−^ mice. Groups of wild-type (WT) and TSLPR^−/−^ mice were infected with 10^3^ *Trypanosoma congolense*. At indicated times, mice were sacrificed, the spleen cells were directly stimulated *ex vivo* with phorbol myristic acetate, brefeldin A and ionomycin for 3–5 h, and their CD4^+^ cells were assessed for intracellular expression of IFN-γ **(A–C)** and TNF-α **(D–F)** by flow cytometry. Shown are contour plots **(A,D)** and bar graphs **(B,C,E,F)** of percentages **(A,B,D,E)** and absolute numbers **(C,F)** of CD4^+^ T cells that express IFN-γ **(A–C)** and TNF-α **(D–F)**. Cells were first gated for CD4 expression and then assessed for IFN-γ and TNF-α expression. Serum levels of IFN-γ **(G)** and TNF-α **(H)** were determined by ELISA. The results presented are representative of 2 different experiments (*n* = 4–5) with similar outcomes. ns, not significant; *p < 0.05; **p < 0.01; ***p < 0.001; ****p < 0.0001.

*Trypanosoma congolense* infection is accompanied by the production of excessive proinflammatory cytokines that contributes to systemic inflammatory response syndrome and death in infected susceptible mice (13, 14). Because TSLPR^−/−^ mice are much more susceptible to infection than their WT counterpart mice (see Figures 1B,C), we hypothesized that the levels of proinflammatory cytokines in TSLPR^−/−^ mice would be significantly higher than in WT mice. Using flow cytometry, we found that the percentages and absolute numbers of IFN-γ (Figures 4A–C) and TNF-α (Figures 4D–F) producing CD4^+^ cells in spleens (Figures 4A–F) and liver (Figures S2A–F in Supplementary Material) of infected TSLPR^−/−^ mice were significantly (*p* < 0.05–0.0001) higher than those of WT control mice, with the difference being more pronounced toward the advanced stage of the infection when TSLPR^−/−^ mice were unable to control parasitemia (see Figure 1B). This was associated with significantly higher serum levels of IFN-γ (Figure 4G) and TNF-α (Figure 4H) in infected TSLPR^−/−^ mice compared to their WT control mice. However, there was no significant difference in the percentages of IL-10-producing CD4^+^ cells in the spleens and liver or serum levels of IL-10 between infected WT and TSLPR^−/−^ mice (Figures S3A–G in Supplementary Material). Interestingly, the absolute numbers of IL-10-producing CD4^+^ cells in infected TSLPR^−/−^ mice were higher than those from WT mice on day 45 (Figures S3C,F in Supplementary Material), and this could be explained by the higher hepatosplenomegaly observed during the late stage of infection (see Figures 1D,E) in TSLPR^−/−^ mice. Collectively, these results show that the absence of TSLP-TSLPR signaling in mice infected with *T. congolense* leads to enhanced systemic production of disease-exacerbating proinflammatory cytokines and this could be a key contributor to the enhanced susceptibility observed in infected TSLPR^−/−^ mice.

### Blockade of IFN-γ in *T. congolense* Infected TSLPR^−/−^ Mice Abolished the Enhanced Susceptibility of Mice and Prolonged Their Survival

**Figure 5:**
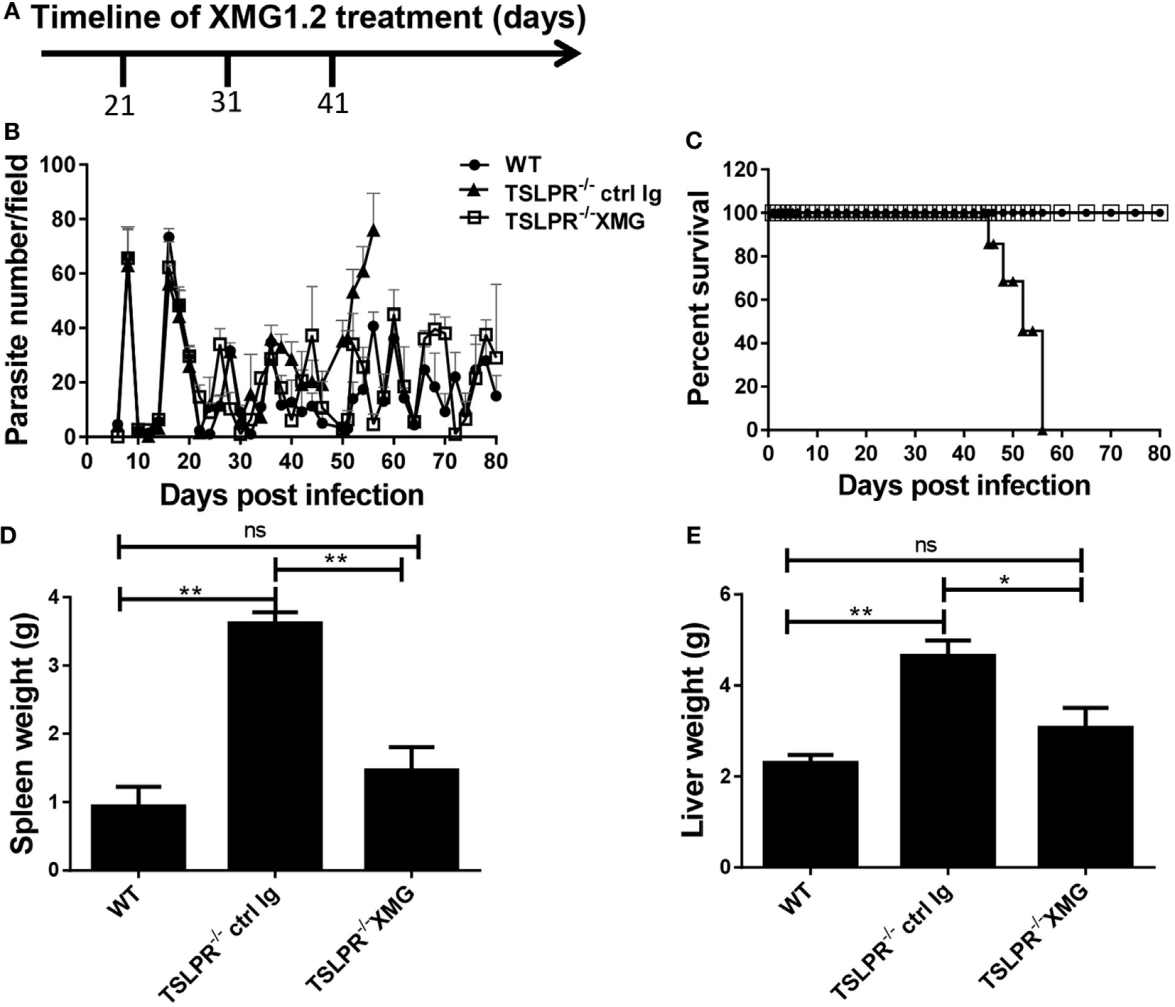
Treatment of TSLPR^−/−^ mice with anti-IFN-γ mAb abolished their enhanced susceptibility to *Trypanosoma congolense* infection. Groups of WT and TSLPR^−/−^ mice were infected intraperitoneally with 10^3^ *T. congolense*, and on indicated days, some TSLPR^−/−^ mice were treated with anti-IFN-γ (XMG1.2, 1mg/mouse) as indicated **(A)**. Daily parasitemia **(B)** and survival **(C)** of infected mice were monitored. 10 days after the last mAb injection, some mice were sacrificed and the spleen **(D)** and liver **(E)** weights were determined. The results presented are representative of two different experiments (*n* = 4–6) with similar outcomes. ns, not significant; *p < 0.05; **p < 0.01.

Because IFN-γ levels were systemically higher in the spleen, liver, and serum of infected TSLPR^−/−^ mice than their WT counterpart mice (Figures 4A–C,G; Figures S2A–C in Supplementary Material), we considered the possibility that high levels of this cytokine may be a major contributor to their enhanced susceptibility and early death. To test this, we infected TSLPR^−/−^ mice with *T. congolense* and on the indicated days (Figure 5A), treated them with anti-IFN-γ mAb, and monitored parasitemia and survival. As shown in Figures 5B,C, infected TSLPR^−/−^ mice treated with anti-IFN-γ controlled several waves of parasitemia during the chronic stage of infection and survived as long as their WT control mice (up to day 80 postinfection when the experiment was terminated). In contrast, infected TSLPR^−/−^ mice treated with control Ig developed uncontrolled parasitemia during the chronic phase of the infection and all succumbed to their infection by day 56 postinfection (Figures 5B,C). Treatment of infected TSLPR^−/−^ mice with anti-IFN-γ mAb was also associated with reduced hepatosplenomegaly (Figures 5D,E). Together, these results indicate that anti-IFN-γ mAb treatment of infected TSLPR^−/−^ mice abolished the enhanced susceptibility to infection, reduced hepatosplenomegaly, and led to prolonged survival of infected TSLPR^−/−^ mice.

### Reduced Level of IFN-γ and TNF-α in Infected TSLPR^−/−^ Mice Treated with Anti-IFN-γ

**Figure 6:**
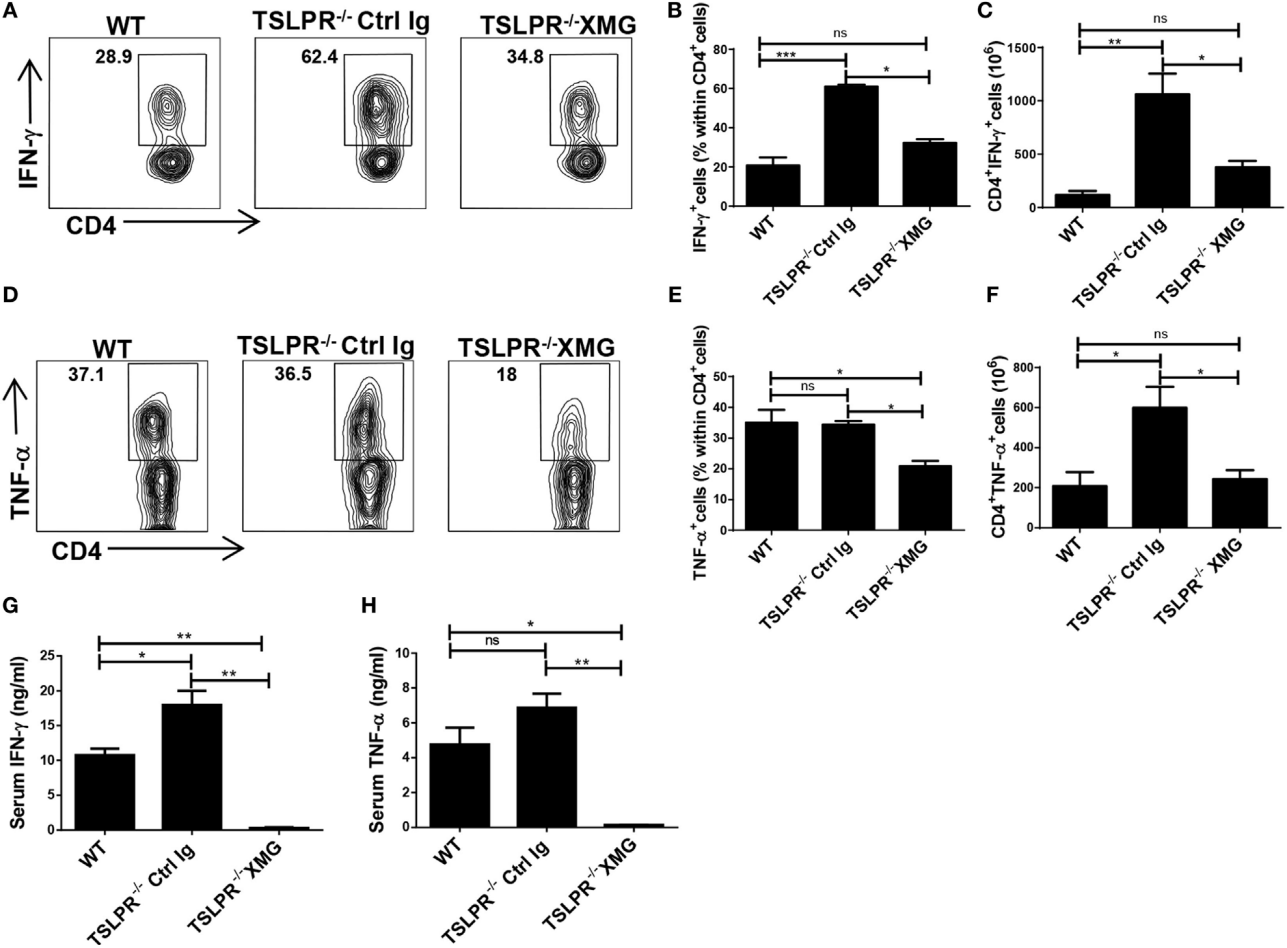
Reduced serum levels and numbers of IFN-γ- and TNF-α-producing CD4^+^ T cells after anti-IFN-γ treatment of *Trypanosoma congolense* infected TSLPR^−/−^ mice. Groups of WT and TSLPR^−/−^ mice were infected intraperitoneally with 10^3^ *T. congolense*. On days 21, 31, and 41 postinfection, some TSLPR^−/−^ mice were treated with anti-IFN-γ mAb and sacrificed on day 10 after the last antibody treatment. Their spleen cells were directly stimulated *ex vivo* with phorbol myristic acetate, brefeldin A, and ionomycin for 3–5 h, and their CD4^+^ cell were assessed for intracellular expression of IFN-γ **(A–C)** and TNF-α **(D–F)** by flow cytometry. Shown are contour plots **(A,D)**, and bar graphs **(B,C,E,F)** of percentages **(A,B,D,E)** and absolute numbers **(C,F)** of CD4^+^ T cells that express IFN-γ **(A–C)** and TNF-α **(D–F)**. Cells were first gated for CD4 expression and then assessed for IFN-γ and TNF-α expression. Serum levels of IFN-γ **(G)** and TNF-α **(H)** were also determined by ELISA. The results presented are representative of two different experiments (*n* = 4–5) with similar outcomes. ns, not significant; *p < 0.05; **p < 0.01; ***p < 0.001.

Since the treatment of infected TSLPR^−/−^ mice with anti-IFN-γ mAb ameliorated the disease and prolonged survival of infected mice (Figures 5B–E), we assessed whether this resulted in changes in the frequency of IFN-γ- and TNF-α-producing CD4^+^ T cells in the spleen and liver as well as serum levels of these cytokines. Infected TSLPR^−/−^ mice treated with anti-IFN-γ mAb showed significant reduction in the percentages and absolute numbers of IFN-γ- (Figures 6A–C) and TNF-α (Figures 6D–F)-producing CD4 cells in the spleens (Figures 6A–F) and liver (Figures S4A–F in Supplementary Material) compared to their control Ig treated and WT groups. This was associated with significant reduction in serum levels of IFN-γ (Figure 6G) and TNF-α (Figure 6H) in the mice treated with anti-IFN-γ compared to their control groups. These results indicate that anti-IFN-γ treatment of *T. congolense*-infected TLSPR^−/−^ mice not only reduced the systemic level of excessive IFN-γ in circulation, it also reduced the levels of TNF-α, an observation that highlights the importance of IFN-γ in driving the activation of TNF-α-producing cells (47).

### Impaired Alternatively Activated Macrophages (M2) in the Spleen and Liver of Infected TSLPR^−/−^ Mice

**Figure 7:**
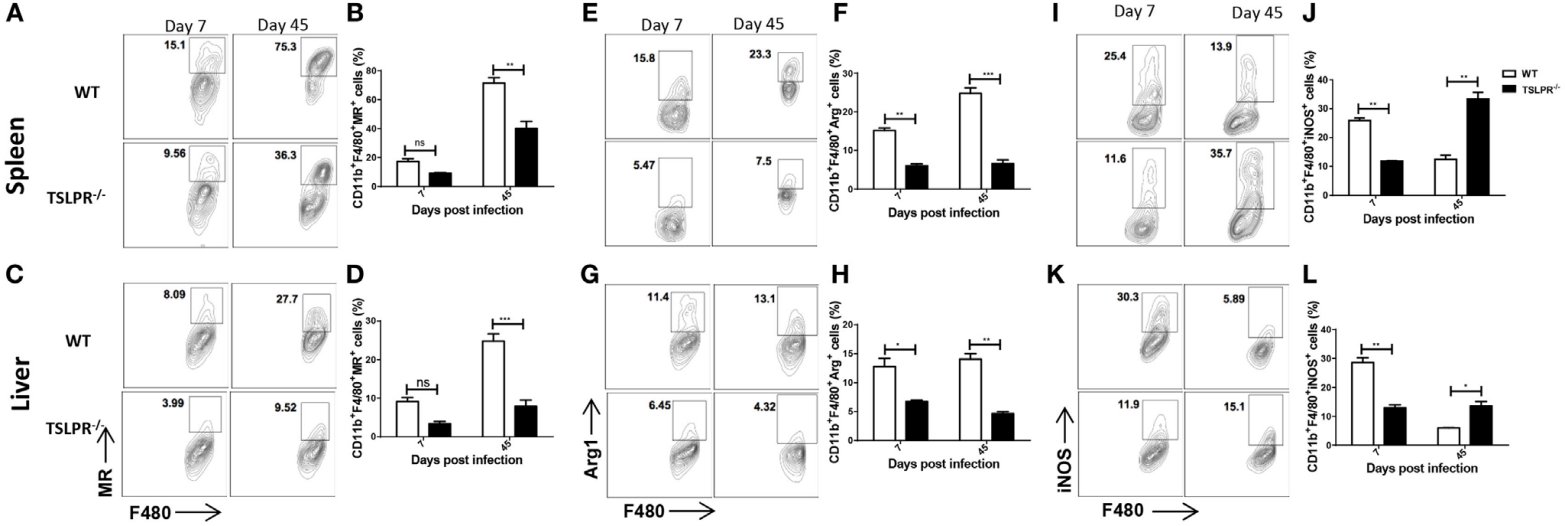
Impaired alternative macrophage (M2) activation in the spleens and liver of infected TSLPR^−/−^ mice. Groups of WT and TSLPR^−/−^ mice were infected with 10^3^ *Trypanosoma congolense*. At indicated times, infected mice were sacrificed, and the spleen and liver cells were stained directly *ex vivo* and assessed for expression of mannose receptor **(A–D)**, Arginase 1 [Arg1, **(E–H)**], and inducible nitric oxide synthase [iNOS, **(I–L)**] on CD11b^+^ F4/80^+^ cells (macrophages) by flow cytometry. The results presented are representative of two different experiments (*n* = 3–4) with similar outcomes. ns, not significant; *p < 0.05; **p < 0.01; ***p < 0.001.

It is known that TSLP enhances the polarization of alternatively activated (M2) macrophages (48, 49). Since high levels of IFN-γ was associated with enhanced susceptibility of TSLPR^−/−^ mice and because M2 macrophages help to dampen excessive M1 driven inflammation (19, 20), we hypothesized that susceptibility of TSLPR^−/−^ mice to *T. congolense* may be related to impaired M2 differentiation in these mice. We infected mice with *T. congolense* and on days 7 and 45 postinfection, assessed the levels of M2 macrophage markers in the spleens and liver by flow cytometry. Mannose receptor (MR) and arginase-1 expression are strongly associated with alternatively activated macrophages (M2) while iNOS is associated with classically activated macrophages (M1) (48, 49). Interestingly, we found significantly reduced expression of MR (Figures 7A–D) and arginase-1 (Figures 7E–H) on CD11b^+^F4/80^+^ cells from the spleens (Figures 7A,B,E,F) and liver (Figures 7C,D,G,H) of TSLPR^−/−^ mice compared to their WT control group. In contrast, the expression of iNOS was significantly increased on macrophages from spleens (Figures 7I,J) and liver (Figures 7K,L) of infected TSLPR^−/−^ mice. Interestingly, WT macrophages showed increased expression of iNOS during the early stages of infection but were able to downregulate this toward the later stage of infection unlike their TSLPR^−/−^ mice (Figures 7I–L). This is consistent with the findings that iNOS contributes to early control of *T. congolense* infection and that a switch from early M1 at the onset to M2 later during infection is beneficial for immunity to African trypanosomes (34, 35). Genes associated with M2 macrophages were also quantified using real time PCR (Figure S5 in Supplementary Material) and were found to be upregulated in the WT mice over TSLPR^−/−^ mice. Collectively, our data show that alternatively activated macrophages, which are regulatory in nature, are impaired in infected TSLPR^−/−^ mice and may be contributing to the observed increase in the aberrant production of disease-promoting proinflammatory cytokines by the CD4^+^ cells.

## Discussion

The main aim of the studies reported here was to investigate the role of thymic stromal lymphopoietin (TSLP), a major cytokine that plays a critical role in optimal type 2 immunity, in experimental *T. congolense* infection in mice. We observed that infection with *T. congolense* induced TSLP expression in C57BL/6 mice, suggesting that this cytokine might play a role in *T. congolense* infection. Using TSLP receptor deficient (TSLPR^−/−^) mice, our results clearly show that the absence of TSLP signaling leads to susceptibility to *T. congolense* infection. This enhanced susceptibility was not related to defective germinal center response in the spleen or changes in numbers of Tregs in infected TSLPR^−/−^ mice. Instead, infected TSLPR^−/−^ mice showed systemic (spleen, liver, and serum) increase in the production of disease-promoting proinflammatory cytokines (IFN-γ and TNF-α) and impaired alternative macrophage activation. Treatment of infected TSLPR^−/−^ mice with anti-IFN-γ led to reduction in splenomegaly, systemic levels of IFN-γ and TNF-α, and abolished the death of infected TSLPR^−/−^ mice. Collectively, these observations show that TSLP-TSLPR engagement is critical for resistance to experimental *T. congolense* infection.

We and others have reported that an optimal germinal center and B cell responses play a crucial role in mediating *T. congolense* control by influencing the quality of protective trypanosome-specific antibodies being produced during infection (5, 50). It is known that both IgM and IgG antibody subclasses are crucial for parasite clearance (51). It was therefore surprising to observe that although infected TSLPR^−/−^ mice were dying significantly earlier than their WT counterpart mice, they did not show significant impairment in serum levels of parasite-specific IgG antibody subclasses (apart from IgG1). This was also further confirmed by the finding of comparable germinal center response and frequencies of germinal center B cells in the spleens of infected WT and TSLPR^−/−^ mice. Paradoxically, despite intact germinal center responses, serum levels of IgG1 in infected TSLPR^−/−^ mice were significantly reduced on day 45 after infection compared to their WT counterpart mice. Previous reports show that IFN-γ is able to suppress IgG1 antibody class switching leading to reduced levels of this antibody isotype (52). It is therefore conceivable that increased production of IFN-γ in TSLPR^−/−^ mice from day 45 after infection could be responsible for reduced serum levels of IgG1 in these mice. This could in turn partly contribute to the enhanced susceptibility of these mice to *T. congolense* during the late phase of infection. In line with this, enhanced survival of *T. brucei*-infection animal has been attributed to increased production of IgG1 in infected mice (35). Of note is the fact that IgG2a and IgG3 were not impaired in TSLPR^−/−^ mice in all the time points assessed.

The numbers of Tregs in the spleens and liver was not different between TSLPR^−/−^ and WT mice throughout infection. Previous reports have shown that Tregs contribute to susceptibility to experimental *T. congolense* infection (10–12), and this was thought to be related to their ability to produce IL-10 (11, 53). There were no differences in both percentages of Tregs in the spleens and liver of infected WT and TSLPR^−/−^ mice. In addition, we found no difference in the serum levels of IL-10 and percentage of IL-10-producing CD4^+^ T cells in these organs. These observations would indicate that IL-10 and Tregs do not contribute to the susceptibility of infected TSLPR^−/−^ mice to *T. congolense* infection.

Classically activated (M1) macrophages are important for parasite control during the early phase of *T. congolense* infection (18, 54). This is often associated with increase in mostly Th1 cytokines (including IFN-γ and TNF-α) which are important for effective clearance of parasites during the early stage of infection (6). However, these cytokines appear to have a double-edge sword effect because they also have been linked with death in susceptible mice (13, 14). Following the initial control of first few waves of parasitemia, there is a strong need for the host to switch from Th1 to Th2 immune response as well as from M1 to M2 macrophages in the later stages of infection (34, 35). This switch to Th2 response and M2 macrophages is critical for regulating excessive inflammatory response necessary to prolong the life of infected animal (37). We found that infected TSLPR^−/−^ mice showed systemic increased levels of IFN-γ and TNF-α and a concomitant increase in the percentages and absolute numbers of CD4^+^ T cells producing these cytokines compared to their WT control mice. This was associated with increased expression of markers associated with M1 macrophage activation and a concomitant decrease in M2 markers on macrophages. Treatment of infected TSLPR^−/−^ mice with anti-IFN-γ mAb systemically reduced IFN-γ and TNF-α levels and rescued the otherwise susceptible TSLPR^−/−^ mice from death due to infection. It is conceivable that the suppression of regulatory M2 macrophages in infected TSLPR^−/−^ mice creates an environment that permits continuous production of proinflammatory cytokines resulting in systemic inflammatory response syndrome and death. Our result is in agreement with previous reports that showed that TSLP is required for optimal M2 macrophage response (48, 49), and further highlights the importance of M2 macrophages in regulating M1 macrophage activation and their associated proinflammatory environment to ensure the survival of infected animals (19, 20).

We found that infected TSLPR^−/−^ mice show increased production of proinflammatory cytokines and this was also associated with impaired parasite clearance during the late phase of infection. This impaired parasite clearance could be related in part to impaired IgG1 response observed in these mice during the late phase of infection. The key question therefore is: which of these two susceptibility factors (increased proinflammatory cytokines and impaired IgG1) is the major cause of death in infected TSLPR^−/−^ mice? In other words, how does one differentiate enhanced susceptibility due pathology associated with excessive inflammatory response from that due to reduced IgG1-dependent impaired parasite control in the TSLPR^−/−^ mice? We clearly demonstrated enhanced inflammatory response and M1 macrophage phenotype during the late phase of infection in TSLPR^−/−^ mice, which has been associated with pathology and death in trypanosome-infected mice (13, 14). Although there was no increase in the levels of liver enzymes in infected TSLPR^−/−^ mice, it is conceivable that high IFN-γ-driven hepatosplenomegaly (55, 56) together with the deleterious inflammatory environment in these mice, could pave the way for various organ dysfunctions, contributing to inefficient parasite control and death (57). Thus, the combination of heightened inflammatory response, excessive classical macrophage activation, and impaired IgG1 response would collectively lead to death in infected TSLPR^−/−^ mice during the late phase of infection.

In conclusion, we have shown that TSLP-TSLPR signaling is critical for resistance of *T. congolense*-infected mice. Mice deficient in TSLPR are highly susceptible to infection and die significantly earlier that their WT control mice. This is associated with systemic increase in proinflammatory cytokine production and impaired M2 macrophage activation. Collectively, our study shows that TSLP-TSLPR engagement is needed in the late stages of infection to create a Th2 environment and optimal M2 macrophage response that would ultimately regulate infection-induced inflammation and prolong survival of infected animals.

## Ethics Statement

The experiments described here were approved by the University of Manitoba Animal Care Committee and carried out in accordance with the regulation of the Canadian Council on Animal Care (Protocol Number 14-014).

## Author Contributions

Design, acquisition, analysis, interpretation of data and critical review: CO, RS, AG, and JU. Drafting: CO. All authors reviewed and approved the manuscript for publication.

## Conflict of Interest Statement

The authors declare that the research was conducted in the absence of any commercial or financial relationships that could be construed as a potential conflict of interest.
