# Supplementary material for: Thymic Stromal Lymphopoietin Is Critical for Regulation of Proinflammatory Cytokine Response and Resistance to Experimental Trypanosoma congolense Infection
Source: Front Immunol. 2017 Jul 14;8:803. doi: 10.3389/fimmu.2017.00803 (PMC5509795; doi:10.3389/fimmu.2017.00803)
Supplement: Supplementary file 1 [file presentation_1.pdf]

Supplementary figures

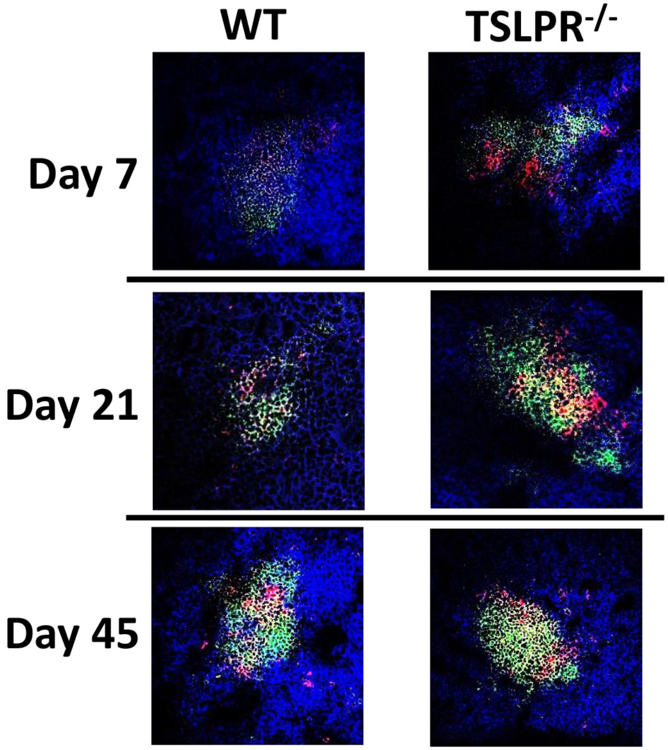

Figure S1. ***Comparable germinal centre formation in the spleen of infected WT and TSLPR$^{-/-}$ mice.*** WT and TSLPR$^{-/-}$ mice were infected with *T. congolense.* At the indicated days, immunofluorescence staining of spleen sections from infected mice were performed. The data presented showed that the germinal centre formation in TSLPR$^{-/-}$ mice was comparable to that of WT mice at both the early and late stages of infection. Data shown are representative of 2 separate experiments (n = 4 mice per experiment) with similar outcome. FITC-labelled PNA, PE anti-CD4 and APC anti-IgD antibodies were used.
