# Supplementary material for: Thymic Stromal Lymphopoietin Is Critical for Regulation of Proinflammatory Cytokine Response and Resistance to Experimental Trypanosoma congolense Infection
Source: Front Immunol. 2017 Jul 14;8:803. doi: 10.3389/fimmu.2017.00803 (PMC5509795; doi:10.3389/fimmu.2017.00803)
Supplement: Supplementary file 2 [file presentation_2.pdf]

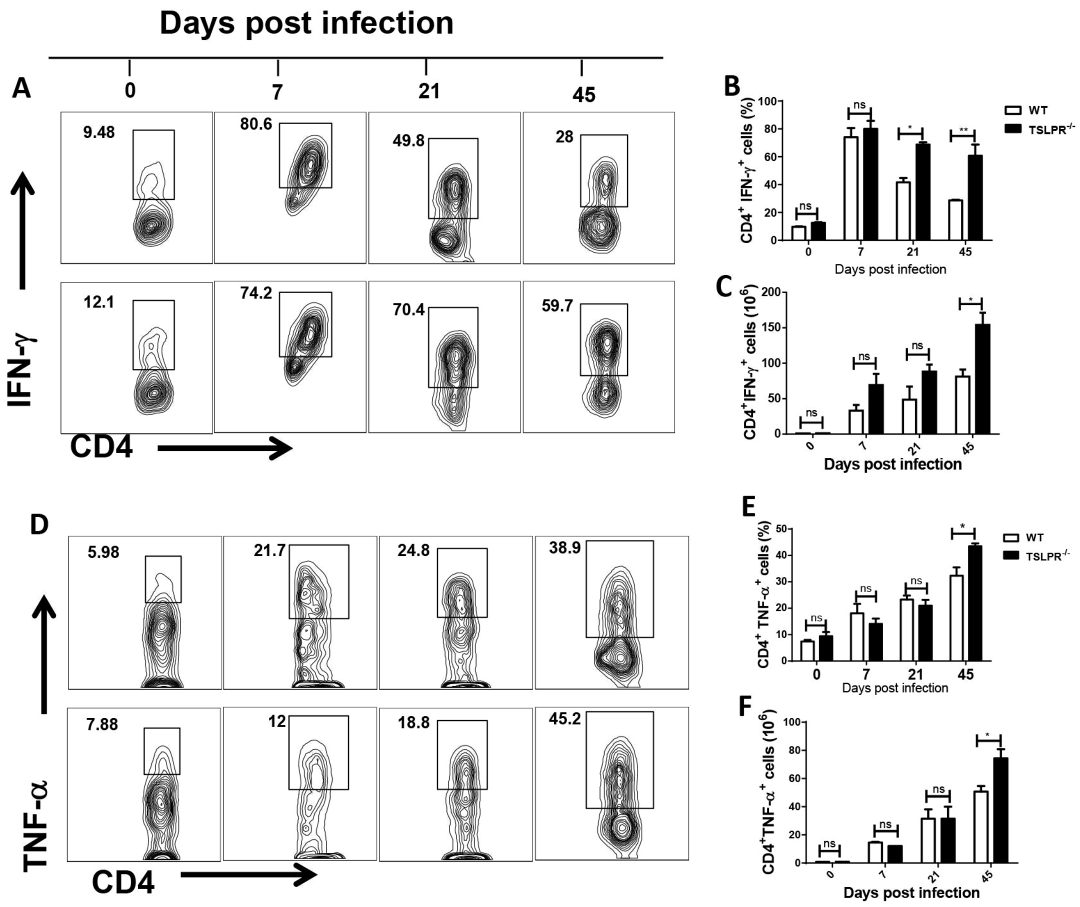

Figure S2. ***Levels of IFN-γ- and TNF-α-producing CD4⁺ T cells in the liver of infected TSLPR⁻/⁻ mice*.**

Groups of WT and TSLPR$^{-/-}$ mice were infected with 10^3 *T. congolense.* At indicated times, mice were sacrificed, the processed liver cells were directly stimulated *ex-vivo* with PMA, BFA and ionomycin for 3-5 hr, and their CD4$^+$ cells were assessed for intracellular expression of IFN-γ (A-C) and TNF-α (D-F) by flow cytometry. Shown are contour plots (A and D), and bar graphs (B, C, E and F) of percentages (A, B, D and E) and absolute numbers (C and F) of CD4$^+$ T cells that express IFN-γ (A-C) and TNF-α (D-F). The results presented are representative of 2 different experiments (n = 4-5) with similar outcomes. ns, not significant; *, $p < 0.05$; **, $p < 0.01$.
