# Supplementary material for: Thymic Stromal Lymphopoietin Is Critical for Regulation of Proinflammatory Cytokine Response and Resistance to Experimental Trypanosoma congolense Infection
Source: Front Immunol. 2017 Jul 14;8:803. doi: 10.3389/fimmu.2017.00803 (PMC5509795; doi:10.3389/fimmu.2017.00803)
Supplement: Supplementary file 3 [file presentation_3.pdf]

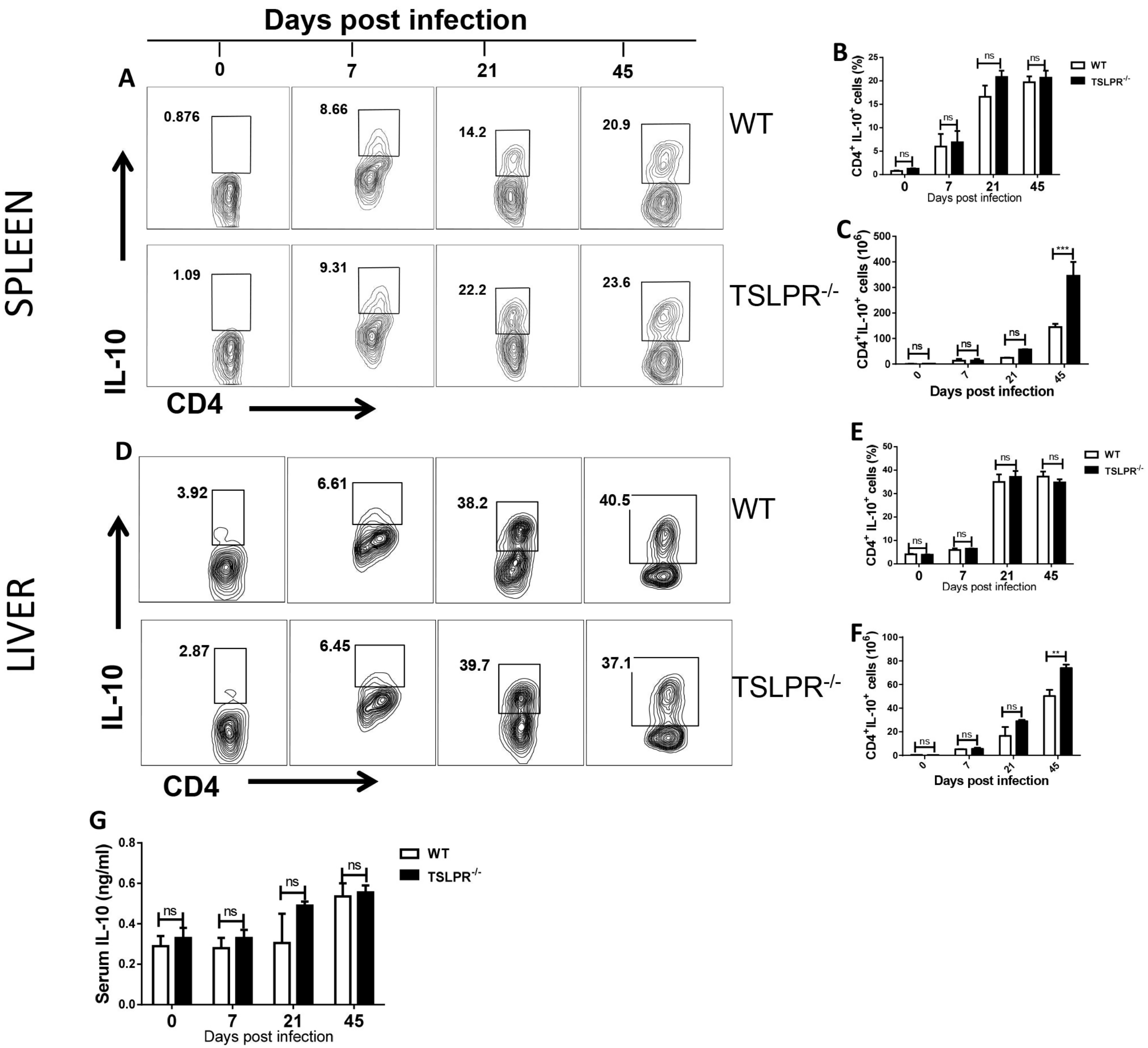

Figure S3. ***Level of IL-10 in the spleen, liver and serum of T. congolense-infected TSLPR$^{-/-}$ mice.***

Groups of WT and TSLPR$^{-/-}$ mice were infected with 10^3 *T. congolense.* At indicated times, mice were sacrificed, the spleen and liver cells were directly stimulated *ex-vivo* with PMA, BFA and ionomycin for 3-5 hr, and their CD4$^+$ cells were assessed for intracellular expression of IL-10 by flow cytometry. Shown are contour plots (A and D), and bar graphs (B, C, E and F) of percentages (A, B, D and E) and absolute numbers (C and F) of CD4$^+$ T cells that express IL-10 (A-F) in the spleen (A-C) and liver (D-F). Serum levels of IL-10 (G) were determined by ELISA. The results presented are representative of 2 different experiments (n = 4-5) with similar outcomes. ns, not significant;; **, $p < 0.01$; ***, $p < 0.001$.
