# Supplementary material for: Thymic Stromal Lymphopoietin Is Critical for Regulation of Proinflammatory Cytokine Response and Resistance to Experimental Trypanosoma congolense Infection
Source: Front Immunol. 2017 Jul 14;8:803. doi: 10.3389/fimmu.2017.00803 (PMC5509795; doi:10.3389/fimmu.2017.00803)
Supplement: Supplementary file 5 [file presentation_5.pdf]

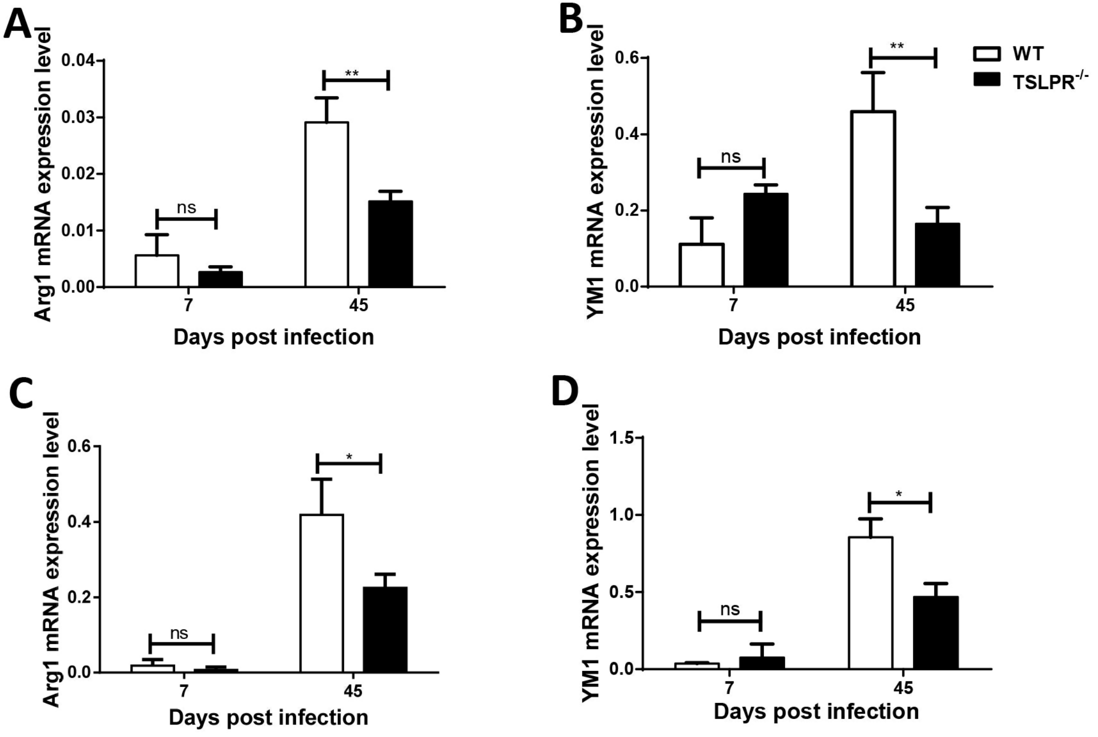

Figure S5. Reduced mRNA levels of alternatively activated macrophages (M2) genes in the spleen and liver of infected TSLPR$^{-/-}$ mice. WT and TSLPR$^{-/-}$ mice were infected with *T. congolense.* At the indicated days, CD11b^{+}F4/80^{+} macrophages were purified. Real-time PCR was performed and the expressions of Arg1 and YM1 analysed. Changes in relative gene expressions were normalized to Eukaryotic Translation Elongation Factor 2 (Eef2). Data shows increased expression of Arginase-1 and YM1 in the spleen (A and B, respectively) and liver (C and D, respectively) of infected mice. Data shown are representative of 2 separate experiments (n = 4 mice per experiment) with similar outcomes.
